# Ethnomedicine, antibacterial activity, antioxidant potential and phytochemical screening of selected medicinal plants in Dibatie district, Metekel zone, western Ethiopia

**DOI:** 10.1186/s12906-024-04499-x

**Published:** 2024-05-21

**Authors:** Baressa Anbessa, Ermias Lulekal, Ariaya Hymete, Asfaw Debella, Eyob Debebe, Abiy Abebe, Sileshi Degu

**Affiliations:** 1 Department of Plant Biology and Biodiversity Management, College of Natural and Computational Sciences, Addis Ababa University, Addis Ababa, Ethiopia; 2 Department of Biology, College of Natural and Computational Sciences, Bule Hora University, Bule Hora, Ethiopia; 3 Department of Pharmaceutical Chemistry and Pharmacognosy, School of Pharmacy, College of Health Sciences, Addis Ababa University, Addis Ababa, Ethiopia; 4 Directorate of Modern and Traditional Medicine Research, Ethiopian Public Health Institute, Addis Ababa, Ethiopia; 5 Traditional and Modern Medicine Research and Development Directorate, Armauer Hansen Research Institute, Addis Ababa, Ethiopia

**Keywords:** Antibacterial, Antioxidant, Phytochemical, Medicinal plants

## Abstract

**Background:**

Medicinal plants play a major role in the delivery of healthcare, particularly among the rural population of Ethiopia. Plant extracts and their bioactive compounds have been utilized for the treatment of several diseases. This study was aimed at evaluating the antibacterial activity, antioxidant capacity, and phytochemical content of selected medicinal plants used in Dibatie district, western Ethiopia.

**Methods:**

Study plants were collected, shade dried, pulverized, extracted by maceration in 80% ethanol, and subjected to antibacterial, antioxidant, and phytochemical tests. Minimum inhibitory concentration (MIC) was determined using 96-well microplates and nutrient broth microdilution. Antioxidant activity was evaluated using the 2,2-diphenyl-1-picrylhydrazyl (DPPH) free radical scavenging assay. Phytochemical screening was conducted using standard test methods.

**Results:**

The ethanolic extract of *Polystachya steudneri* Rchb.f. pseudobulbs was the most active against gram-negative *Proteus mirabilis*, *Salmonella typhimurium*, *Klebsiella pneumoniae*, *Escherichia coli*, and *Shigella flexneri*, with MIC values of 8 ± 0, 11 ± 5, 3 ± 1, 3 ± 1, and 2 ± 0 mg/mL, respectively. The ethanolic extract of *P. steudneri* was also the most effective against gram-positive *Staphylococcus aureus*, *Staphylococcus epidermidis*, *Streptococcus agalactiae*, and *Enterococcus faecalis*, with MIC values of 8 ± 0, 8 ± 0, 3 ± 1, and 16 ± 0 mg/mL, respectively. Ethanolic extracts of *Gnidia involucrata* Steud. ex A.Rich. stems and roots were effective antioxidants, with respective 50% DPPH free radical inhibitory concentrations (IC_50_) of 168.68 and 181.79 µg/mL, followed by that of *P. steudneri* (IC_50_ = 203.11 µg/mL). The study plants contained alkaloids, anthocyanins, anthraquinones, cardiac glycosides, coumarins, flavonoids, phenols, saponins, steroids, tannins, and terpenoids.

**Conclusions:**

This study confirmed the antibiotic, antioxidant, and phytochemical constituents of the investigated plants and suggested further investigations that may lead to bioactive lead compounds.

## Background

Infectious diseases are the most common causes of mortality and morbidity among human beings throughout the world [1]. Recently, the rapid emergence and spread of multidrug-resistant pathogens have been considered major challenges for the treatment of several infectious diseases [2, 3]. The multiple drug resistance mechanisms include drug uptake limitation, drug target modification, drug inactivation, and active drug efflux, and the resistance processes differ based on microbial types [4]. Besides, certain pathogenic bacteria form biofilms through quorum sensing and develop drug resistance [5]. Hence, there is a need for the discovery of new drugs against multidrug-resistant pathogenic microorganisms.

Plant extracts and their bioactive compounds have been utilized for the treatment of several diseases since ancient times [5]. Medicinal plants were the major sources of bioactive compounds that could be used as potential alternatives to conventional antimicrobials [6]. The antibacterial activity of the plants could be ensured either by inhibiting the growth of bacteria or by disturbing the cell-to-cell communication system between the bacteria through anti-quorum sensing (AQS) [7], in which the latter is currently preferable, especially against antimicrobial-resistant bacteria. Hence, plant-derived medicines have been considered convenient therapies due to their fewer side effects and greater pharmacological efficacy [8]. Medicinal plants contain natural phytochemicals such as alkaloids, flavonoids, saponins, tannins, terpenoids, steroids, resins, cardiac glycosides, coumarins, and phenolic compounds, among others, that could have a multitude of biological activities [9].

Moreover, medicinal plants are potent antioxidants and play an important role in sequestering reactive oxygen species (ROS) in living cells owing to the presence of various phytochemicals [10]. Polyphenols from plants scavenge free radicals and inhibit enzymes that are responsible for the formation and accumulation of reactive oxygen species (ROS) [11]. Antioxidant phenolic compounds reduce the level of free radicals in living cells, thereby preventing the oxidation of cellular components by donating hydrogen atoms to free radicals and forming stable, nontoxic compounds like phenoxyl radicals [12]. These compounds prevent or treat diseases related to oxidative stress, such as cancer, diabetes, cardiovascular diseases, inflammatory joint diseases, dementia, asthma, eye diseases, and atherosclerosis [10]. Additionally, plant-derived phenolic compounds capture and neutralize free radicals in human cells to protect them from aging [13].

Ethiopia is a center for plant diversity, diverse topography, and multiple ethnic groups, languages, cultures, and beliefs, which enhance the practice of using medicinal plants. Particularly, the Metekel zone in Benishangul Gumuz Regional State has various ethnic groups (e.g., Agaw, Amhara, Gumuz, Oromo, and Shinasha), multiple cultures, and a diversity of medicinal plants. For instance, *Asparagus flagellaris* (Kunth) Baker, *Brucea antidysenterica* J. F. Mill., *Celosia trigyna* L., *Crepis rueppellii* Sch. Bip., *Gnidia involucrata* Steud. ex A.Rich., *Polystachya steudneri* Rchb.f., and *Sauromatum venosum* (Aiton) Kunth are traditionally used to treat toothache, leishmaniasis, tapeworm, diarrhea, gonorrhea, wounds, and amoeba, respectively, in the Dibatie district of the Metekel zone, western Ethiopia. However, there are limited reports yet on the ethnomedicine, antimicrobial activity, antioxidant properties, and phytochemical profiles of these plants in Ethiopia as a whole and in the Dibatie district of the Metekel zone in particular. Therefore, the current study was aimed at evaluating the antibacterial activity, antioxidant potential, and phytochemical constituents of the above medicinal plants.

## Methods

### Study period, study design and area

This study involved a preliminary ethnomedicinal survey through a semi-structured interview [14], which was conducted from April 2021 to June 2022. Following the ethnomedicinal investigation, the medicinal plants were selected, and the laboratory samples were collected in November 2022. Then, the laboratory work was carried out from February to June 2023. The field ethnobotanical data and sample collection were conducted in the Dibatie district of the Metekel zone, Benishangul Gumuz Regional State, western Ethiopia. Residents from eleven kebeles (sub-districts) such as Berber, Dibatie-02, Donben, Galessa, Gipho, Jan, Lega-buna, Parzeyit, Qorqa, Sombo-sire, and Tuski-gambela participated in the interview process. The plant material preparation, extraction, antibacterial test, antioxidant assay, and phytochemical screening were carried out at the laboratory of the Directorate of Modern and Traditional Medicine Research at the Ethiopian Public Health Institute, Addis Ababa, Ethiopia.

### Plants selection

Medicinal plants were selected for laboratory work depending on the prior ethnobotanical survey of their traditional medicinal uses. The selection criteria were mainly based on the medicinal plants traditionally used to treat human ailments such as amoeba, diarrhea, gonorrhea, leishmaniasis, tapeworm, toothache, and wounds. Because these diseases were caused or aggravated due to the infestation by bacteria, protozoans, or helminthes. The selection of the study plants also emphasized the relative curing potential of each plant species (percentage of fidelity level) to heal the above ailments. The percentage of fidelity level helps to give a hint for further investigations on the medicinal efficacy of bioactive constituents. The percentage of fidelity level (FL%) was calculated using the formula: FL% = Ip/Iu × 100, where Ip is the number of respondents who indicated the use of a species for the same major ailments and Iu is the total number of respondents who mentioned the plant for any major ailments indicated [15]. Accordingly, plants with a fidelity level greater than 45% were selected for the laboratory investigations (Table 1). In addition, the selected plants were prioritized since they were not studied using the same methods as the present study so far.

**Table 1:** Ethnomedical data on medicinal plants selected for laboratory based studies

Scientific name	Family	Local name (Language)	Geographic location	Ailments treated	Parts used	Ip	Iu	FL%	Voucher number
*Asparagus flagellaris*	Asparagaceae	Saritii (AO)	10˚34.745’N, 36˚11.095’E	Toothache	Root	4	7	57	BA-04
*Brucea antidysenterica*	Simaroubaceae	Qomonyoo (AO)	10˚15.582’N, 36˚05.918’E	Leishmaniasis	Fruit	5	10	50	BA-45
*Celosia trigyna*	Amarnthaceae	Babilinda (AO)	10˚26.480’N, 36˚08.175’E	Tapeworm	Inflorescence, seed	10	10	100	BA-77
*Crepis rueppellii*	Asteraceae	Yefyel wetet (Am)	10˚33.849’N, 36˚07.970’E	Diarrhea	Root	5	9	56	BA-62
*Gnidia involucrata*	Thymelaeaceae	Qamaxxee (AO)	10˚34.235’N, 36˚11.879’E	Gonorrhea	Root	6	13	46	BA-151
*Polystachya steudneri*	Orchidaceae	-	10˚29.878’N, 36˚10.399’E	Wounds	Pseudobulb	6	12	50	BA-08
*Sauromatum venosum*	Araceae	Muuna (Sh)	10˚36.015’N, 36˚10.102’E	Amoeba	Tuber	4	8	50	BA-42

*AO* Afaan Oromoo, *Am* Amharic, *Sh* Shinashigna

### Sample collection and preparation

Based on the above criteria, roots of *Asparagus flagellaris*, fruits of *Brucea antidysenterica*, inflorescence having seeds of *Celosia trigyna*, roots of *Crepis rueppellii*, roots and stems of *Gnidia involucrata*, pseudobulbs of *Polystachya steudneri*, and tubers of *Sauromatum venosum* were collected from Berber, Galessa, Jan, Lega-buna, Sombo-sire, and Tuski-gambela sub-districts. The collected plants were identified by Dr. Ermias Lulekal and Mr. Baressa Anbessa in the department of plant biology and biodiversity management at Addis Ababa University. In addition, the scientific names were checked by referring to the website Plants of the World Online (POWO). Voucher specimens were deposited at the National Herbarium of Addis Ababa University (ETH).

Fruits, seeds, and inflorescences of the indicated plants (Table 1) were shade dried without washing since their dust content was negligible. Roots, stems, and pseudobulbs were washed with tap water, rinsed with distilled water to remove dust, and shade dried in a solar drier. Dried samples were pulverized using an electric grinder to a moderately fine powder and kept in the refrigerator at 4 ºC until extraction.

### Extraction process

As the local community usually uses water as a solvent, aqueous 80% ethanol was used for effective extraction of bioactive compounds from medicinal plant materials. The reason is that aqueous-alcoholic (80% ethanol) extracts are better in phytochemical (e.g., phenolics, flavonoids, tannins, etc.) content and antioxidant activity [16, 17]. The extraction was carried out by macerating 50 g of powdered plant parts in 500 mL of 80% ethanol and continuously shaking for 24 h using a magnetic stirrer. The mixture was filtrated using Whatman number 1 filter paper. The residue was re-macerated for 24 h and filtered. The filtrates were combined and concentrated *in vacuo* using a rotary evaporator (BUCHI R-300 Rotavapor, Switzerland). The concentrated extracts were dried in a water bath at 40 ºC and kept in desiccators with active silica gel until they dried well.

### Antibacterial assay

The test microorganisms were from the American Type Culture Collection (ATCC). Ethanolic extracts of each sample were tested in vitro against the active pathogenic bacterial strains existing in the laboratory. These include the gram-negative (*Proteus mirabilis* ATCC-35,659, *Salmonella typhimurium* ATCC-13,311, *Klebsiella pneumoniae* ATCC-700,603, *Escherichia coli* ATCC-25,922, and *Shigella flexneri* ATCC-12,022) and the gram-positive (*Staphylococcus aureus* ATCC-25,923, *Staphylococcus epidermidis* ATCC-12,228, *Streptococcus agalactiae* ATCC-12,386, and *Enterococcus faecalis* ATCC-1,829,212) bacterial strains.

Nutrient broth and Mueller-Hinton agar were used for microorganism sub-culturing and growing. For that purpose, 13 g of nutrient broth was dissolved in 1000 mL of distilled water, well mixed, and autoclaved at 121 °C and 15 pounds per square inch (psi) for 15 min. Mueller-Hinton agar (38 g) was also dissolved in 1000 mL of distilled water, well mixed, boiled on a hot plate, and autoclaved.

Mueller-Hinton agar for bacteria was used for the subculturing of microorganisms. In this regard, 3–5 well-isolated colonies of the same morphological type from the refreshed agar plate culture were selected. The bacterial colonies were inoculated on sterilized plates containing Mueller-Hinton agar, followed by incubation at 37 °C for 24 h. Later, the bacterial colonies were transferred to the nutrient broth using the sterilized inoculating loop.

The minimum inhibitory concentration (MIC) was determined using 96-well microplates by the nutrient broth microdilution method. Tween 80 was used to dissolve the extracts since it is a low-toxicity surfactant that increases the solubility of bioactive phytochemicals [18]. The ethanolic extract of each sample was dissolved in 5% Tween 80 to an end concentration of 32 mg/mL, which needs to be engaged in serial dilutions. An aliquot of 100 µL of each extract was subjected to serial dilutions in nutrient broth to concentrations of 16, 8, 4, 2, 1, 0.50, 0.25, and 0.13 mg/mL. A standard reference (ciprofloxacin) was taken as a positive control in concentrations of 10, 5, 2.50, 1.25, 0.63, 0.31, 0.16, and 0.08 µg/mL. Tween 80 (5%) was used as a negative control. The microorganism suspension was standardized to 1 × 10^8^ CFU/mL (0.08 to 1.00 turbidity) using a UV-Vis spectrophotometer at 625 nm. An aliquot of 100 µL of standardized microorganisms was inoculated into each well containing serially diluted extracts and controls (positive, negative, and growth controls), except for sterility control. Then, plates were incubated at 37 °C for 18–24 h. In order to read the microorganism growth, 40 µL of 2, 3, 5 tripenyl tetrazolium chloride (TTC) with a concentration of 0.40 mg/mL was added into each well and incubated at 37 °C for 30 min. The development of pink color in the microplate well indicated the presence of living cells (microorganisms), and the reverse result showed inhibition of microbial growth. The lowest concentration of each extract displaying no visible pink color was recorded as the MIC.

During the antibacterial test, we followed various safety practices to avoid any potential hazards. Bacterial cultures were treated as potential pathogens. All materials, media, tubes, plates, loops, needles, pipettes, and other items used were sterilized by autoclaving or using commercially sterilized products. Work spaces were thoroughly cleaned using 70% ethanol or 10% bleach both before and after usage. Mouth pipetting was avoided by staying away from food and drink in the laboratory and washing hands with disinfectant soap before and after working. Labeling everything clearly, autoclaving or disinfecting all waste material, and cleaning up spills with care were also the other safety precautions that we followed during the experiments. Additionally, all necessary personal protective equipment and biological safety cabinets (class II) were used to avoid contamination.

### Antioxidant (2,2-diphenyl-1-picrylhydrazyl (DPPH)) assay

The free radical scavenging ability of ethanolic extracts was determined by using a 2,2-diphenyl-1-picrylhydrazyl (DPPH) assay [19]. Briefly, a fresh 0.1 mM DPPH solution was prepared in 80% ethanol. Ethanolic extracts and ascorbic acid (a positive control) were kept in test tubes at different concentrations (15.63–500 µg/mL) through serial dilution in 80% ethanol. Then, 1 mL of DPPH solution was mixed with 1 mL of each extract and a positive reference in the test tube. The mixtures were shaken thoroughly and incubated in the dark for 30 min at room temperature. The mixture of 1 mL of 80% ethanol and 1 mL of DPPH solution was considered a blank. The absorbance of each mixture was measured at 517 nm against a blank using a UV-VIS spectrophotometer (UV-1800 SHIMADZU).

The percentage of inhibition was calculated using the formula: % Inhibition = [(Ab - As) / Ab] x 100, where Ab is the absorbance of the blank and As is the absorbance of the sample. Later, the 50% inhibition concentration (IC_50_) was calculated for ascorbic acid and extracts of medicinal plants by using the slope equation: Y = mx + c [10].

### Phytochemical screening

The ethanolic extracts were employed for preliminary screening of phytochemicals such as alkaloids, anthocyanins, anthraquinones, cardiac glycosides, coumarins, flavonoids, phenols, saponins, steroids, tannins, and terpenoids following the standardized protocols [5, 13, 20–22]. The results were expressed as (+) for the presence and (-) for the absence of phytochemical compounds.

### Statistical data analysis

The percentage of fidelity level was computed based on the ethnobotanical data to assess the healing potential of each plant species against the corresponding disease. The minimum inhibitory concentration (MIC) data were described as the means ± standard deviation of triplicate analyses. Depending on the MIC values, the principal component analysis (PCA) was computed to indicate variations in the antibiotic effect of medicinal samples and the susceptibility of bacterial strains using R-statistical packages (ggplot2 and grid). The DPPH free radical scavenging activity and the 50% inhibition concentration (IC_50_) were expressed as means of triplicate determinations. Qualitative phytochemical profiles were expressed as the presence (+) and absence (-) of phytochemical constituents. Microsoft Excel version 2013 was also used for the data analysis.

## Results

### Antibacterial activities of medicinal plants

Minimum inhibitory concentration (MIC) values of selected medicinal plants were evaluated against gram-negative (*Proteus mirabilis*, *Salmonella typhimurium*, *Klebsiella pneumoniae*, *Escherichia coli*, and *Shigella flexneri*) and gram-positive (*Staphylococcus aureus*, *Staphylococcus epidermidis*, *Streptococcus agalactiae*, and *Enterococcus faecalis*) bacterial strains at concentrations less than or equal to 16 mg/mL.

### MIC values of plant extracts against gram-negative bacteria

The ethanolic extract of *P. steudneri* pseudobulbs showed the highest antibacterial activity against gram-negative bacterial strains by inhibiting *P. mirabilis*, *S. typhimurium*, *K. pneumoniae*, *E. coli*, and *S. flexneri* with MIC values of 8 ± 0, 11 ± 5, 3 ± 1, 3 ± 1, and 2 ± 0 mg/mL, respectively. Whereas the ethanolic extract of *A. flagellaris* roots exhibited the lowest antibacterial activity as it inhibited both *S. typhimurium* and *E. coli* at MIC values of 16 ± 0 mg/mL, and *P. mirabilis, K. pneumoniae*, and *S. flexneri* at MIC values > 16 mg/mL each (Table 2).

**Table 2:** MIC values of 80% ethanolic extracts (mg/mL) of studied medicinal plants and ciprofloxacin (µg/mL) against gram-negative bacterial strains

Plant species and controls	Bacterial strains				
	*P*. mirabilis	S. typhimurium	K. pneumoniae	E. coli	S. flexneri
*A. flagellaris* (root)	> 16	16 ± 0	> 16	16 ± 0	> 16
*B. antidysenterica* (fruit)	> 16	16 ± 0	> 16	> 16	8 ± 0
*C. trigyna* (seed, inflorescence)	16 ± 0	8 ± 0	13 ± 5	7 ± 2	13 ± 5
*C. rueppellii* (root)	> 16	8 ± 0	16 ± 0	8 ± 0	16 ± 0
*G. involucrata* (root)	> 16	8 ± 0	16 ± 0	8 ± 0	8 ± 0
*G. involucrata* (stem)	> 16	8 ± 0	8 ± 0	8 ± 0	8 ± 0
*P. steudneri* (pseudobulb)	8 ± 0	11 ± 5	3 ± 1	3 ± 1	2 ± 0
*S. venosum* (tuber)	> 16	> 16	> 16	8 ± 0	> 16
Ciprofloxacin	< 0.08	0.31	0.31	< 0.08	1.25
Tween 80	–	–	–	–	–

Values are means ± standard deviation of triplicate examinations

### MIC values of plant extracts against gram-positive bacteria

The ethanolic extracts of *P. steudneri* pseudobulbs and *G. involucrata* stems were the most active against gram-positive bacterial strains. The extract of *P. steudneri* inhibited *S. aureus*, *S. epidermidis*, *S. agalactiae*, and *E. faecalis* at MIC values of 8 ± 0, 8 ± 0, 3 ± 1, and 16 ± 0 mg/mL, and that of *G. involucrata* stems inhibited these bacteria at MIC values of 3 ± 1, 16 ± 0, 2 ± 0, and 16 ± 0 mg/mL, respectively. On the other hand, ethanolic extracts of *A. flagellaris* roots and *S. venosum* tubers were active only against *S. agalactiae*, with MIC values of 4 ± 0 and 2 ± 0 mg/mL, respectively (Table 3).

**Table 3:** MIC values of 80% ethanolic extracts (mg/mL) of studied medicinal plants and ciprofloxacin (µg/mL) against gram-positive bacterial strains

Plant species and controls	Bacterial strains			
	S. aureus	S. epidermidis	S. agalactiae	E. faecalis
*A. flagellaris* (root)	> 16	> 16	4 ± 0	> 16
*B. antidysenterica* (fruit)	4 ± 0	> 16	8 ± 0	16 ± 0
*C. trigyna* (seed, inflorescence)	16 ± 0	> 16	16 ± 0	8 ± 0
*C. rueppellii* (root)	4 ± 0	> 16	4 ± 0	> 16
*G. involucrata* (root)	3 ± 1	> 16	2 ± 0	> 16
*G. involucrata* (stem)	3 ± 1	16 ± 0	2 ± 0	16 ± 0
*P. steudneri* (pseudobulb)	8 ± 0	8 ± 0	3 ± 1	16 ± 0
*S. venosum* (tuber)	> 16	> 16	2 ± 0	> 16
Ciprofloxacin	0.63	0.63	0.63	1.25
Tween 80	–	–	–	–

Values are means ± standard deviation of triplicate examinations

### Coordinates of study plants and bacterial strains based on MIC values

**Fig. 1:**
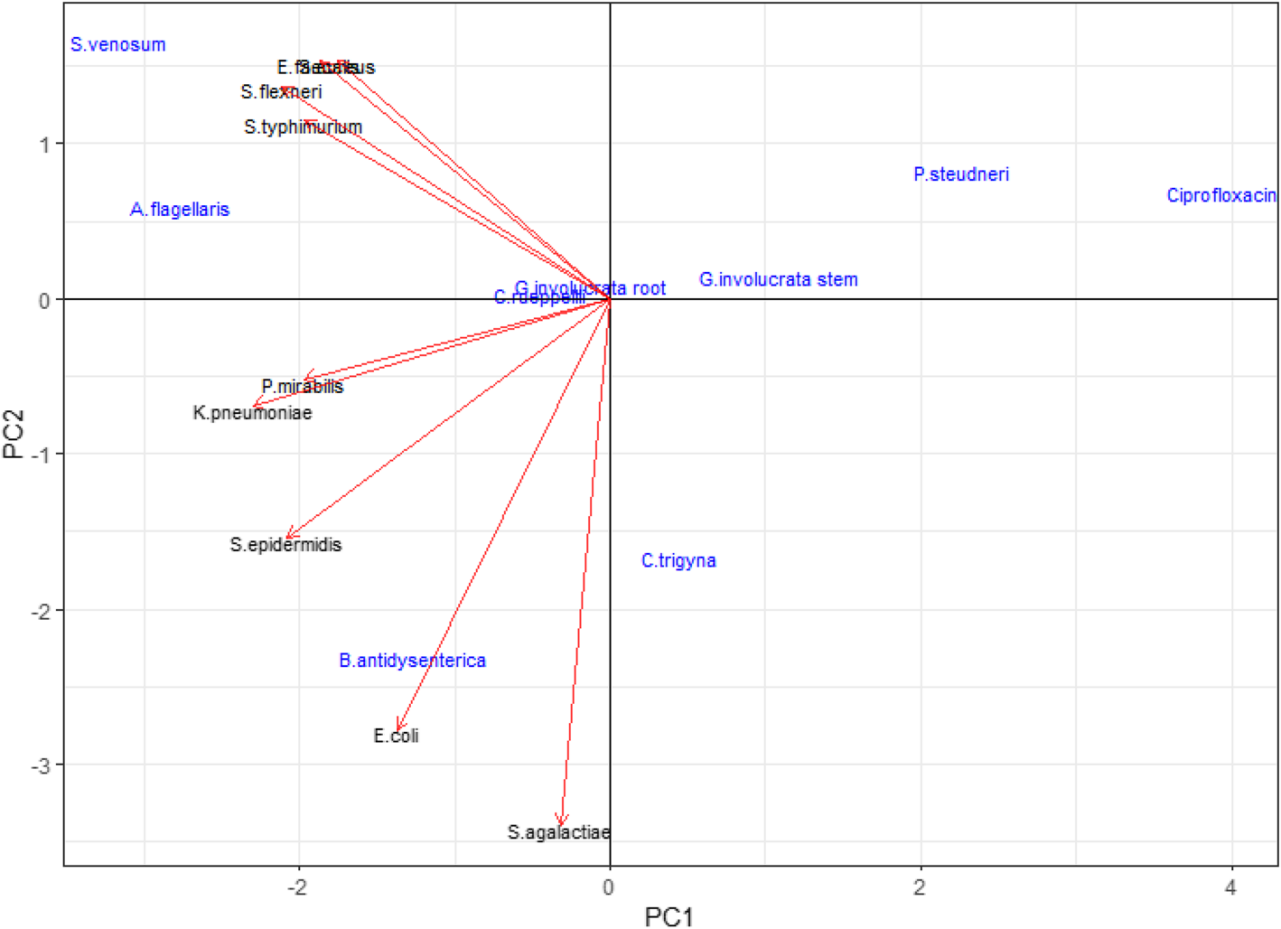
The PCA illustrating the positions of medicinal samples based on their MIC values against the tested bacterial strains

The results of principal component analysis (PCA) revealed that the ethanolic extract of *P. steudneri* pseudobulb was the most effective antibacterial, closest to the positive reference (ciprofloxacin). The extracts of *A. flagellaris* root and *S. venosum* tuber were the least effective antibacterials among the studied medicinal plant species. The results of PCA also showed that *S. agalactiae* was the most susceptible bacterial strain, illustrated with the longest arrow, while *P. mirabilis* was found to be the least susceptible (Fig. 1).

### Antioxidant activity of the study plants

#### DPPH free radical scavenging ability

**Fig. 2:**
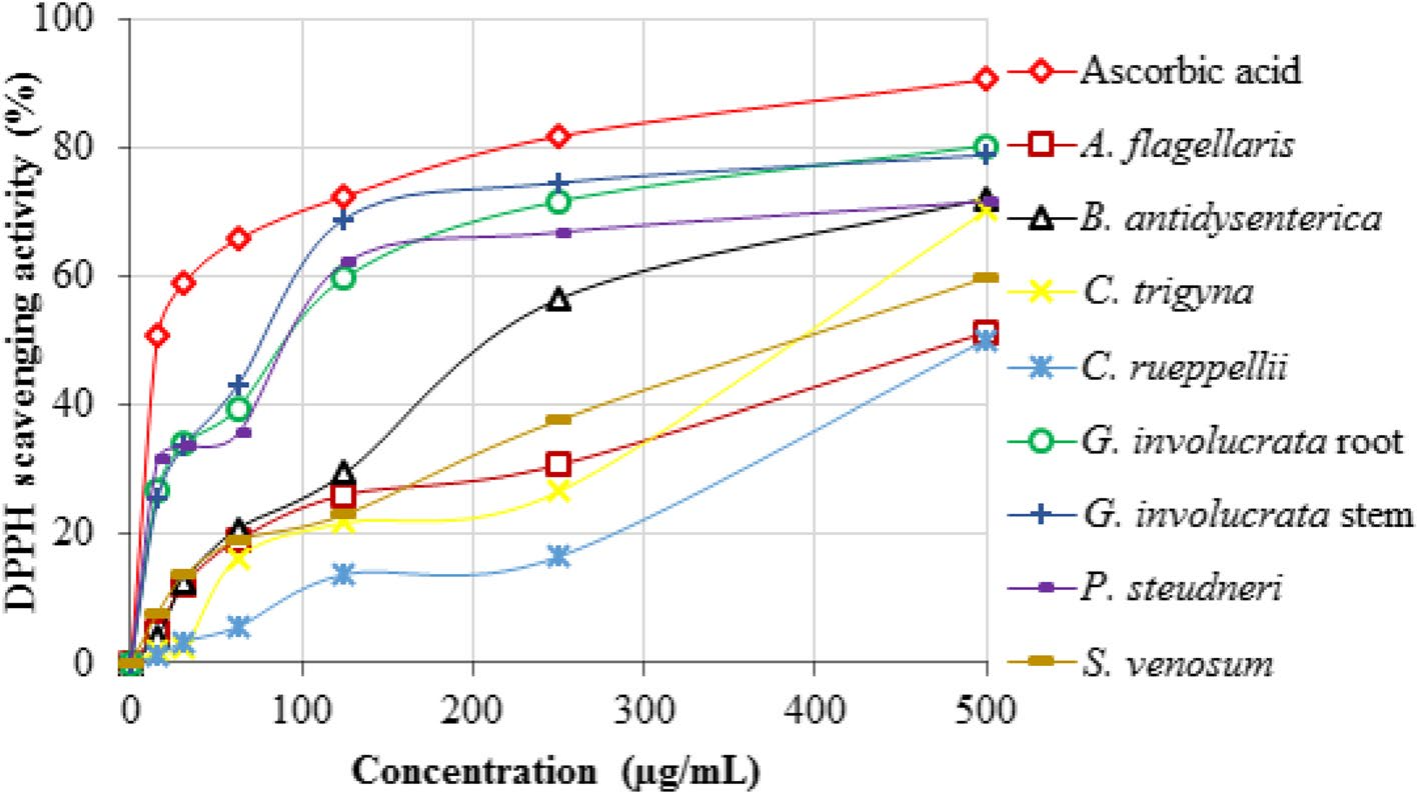
Percentage of DPPH free radical scavenging activity

The deep purple color of the DPPH solution changed to colorless when it was mixed with plant extracts having antioxidant properties and ascorbic acid (positive reference). In contrast, the purple color was retained when the DPPH solution was mixed with extracts of plants with less antioxidant activity and the negative control. In this perspective, following the ascorbic acid, ethanolic extracts of *G. involucrata* stems and roots exhibited the highest DPPH free radical scavenging activity, while extracts of *C. rueppellii* roots showed the lowest (Fig. 2).

#### The 50% inhibitory concentration (IC_50_)

**Fig. 3:**
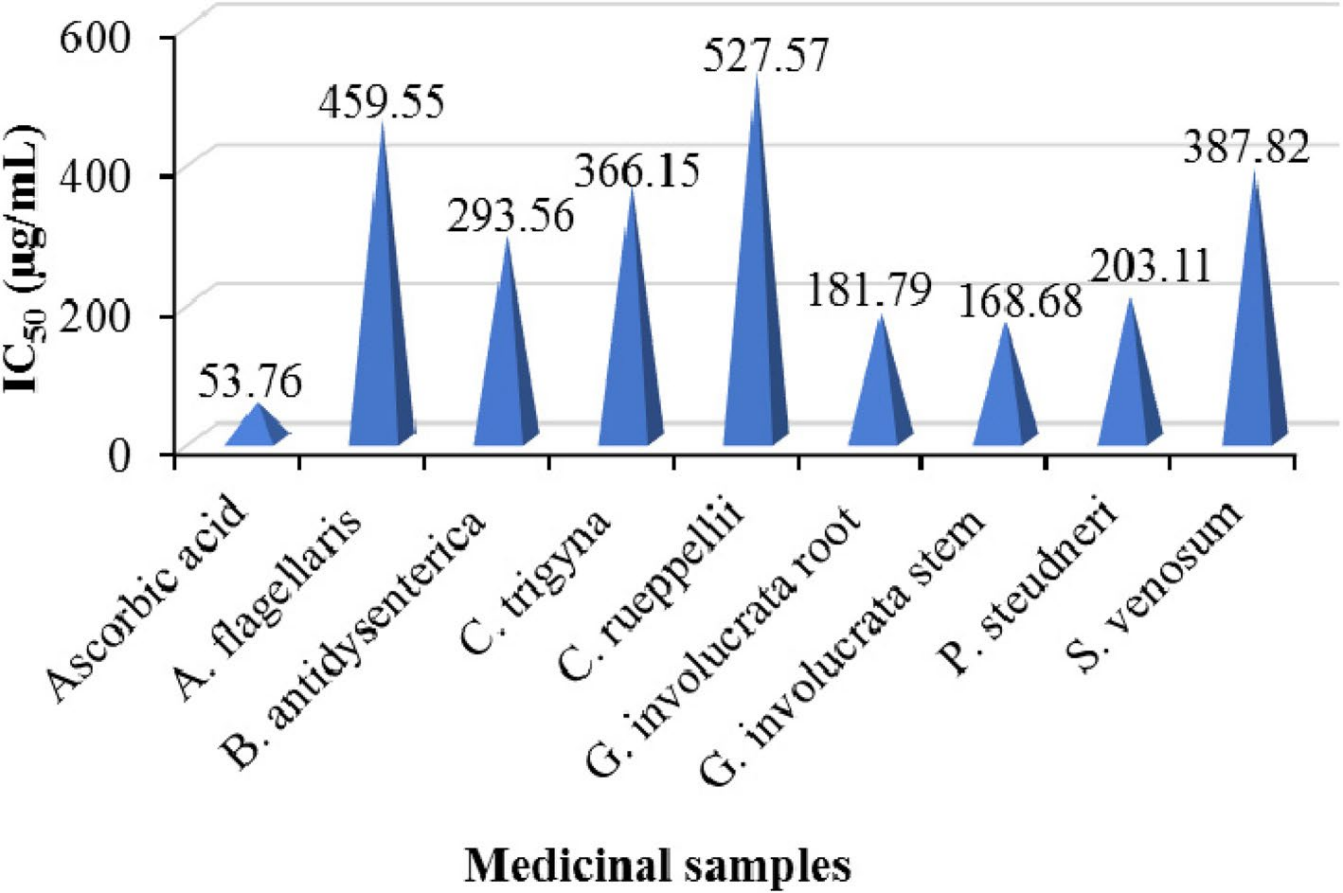
50% inhibition concentration (IC_50_) of the medicinal samples

Relative to the positive reference (IC_50_ = 53.76 µg/mL), ethanolic extracts of *G. involucrata* stems showed IC_50_ value of 168.68 µg/mL, followed by extracts of *G. involucrata* roots (IC_50_ = 181.79 µg/mL). Ethanolic extracts of the remaining plants, such as *P. steudneri, B. antidysenterica, C. trigyna, S. venosum, A. flagellaris*, and *C. rueppellii*, exhibited IC_50_ values of 203.11, 293.56, 366.15, 387.82, 459.55, and 527.57 µg/mL, respectively (Fig. 3).

#### Phytochemical screening of medicinal plants

Qualitative phytochemical screening was carried out to determine the presence or absence of alkaloids, anthocyanins, anthraquinones, cardiac glycosides, coumarins, flavonoids, phenols, saponins, steroids, tannins, and terpenoids in the ethanolic extracts of the studied medicinal plants, and a summary of the findings is presented in Table 4. Accordingly, root extracts of *A. flagellaris* were confirmed to have the tested compounds apart from anthraquinones and steroids. Extracts of *B. antidysenterica* fruits contained all the other tested phytoconstituents except anthocyanins, anthraquinones, cardiac glycosides, and saponins. Except for anthraquinones, the tested phytochemicals were detected in extracts of *C. trigyna* inflorescence with seeds. The root extracts of *C. rueppellii* were observed to contain alkaloids, cardiac glycosides, coumarins, and terpenoids. Extracts of *G. involucrata* roots and stems contained almost similar phytochemicals (anthocyanins, anthraquinones, cardiac glycosides, flavonoids, phenols, tannins, and terpenoids) except for the presence of saponins in roots but not in stems, and the reverse was true for steroids. The tested phytochemicals were observed in the extracts of *P. steudneri* pseudobulbs, except for alkaloids, saponins, and terpenoids. Tubers of *S. venosum* contained the other tested constituents except anthocyanins, anthraquinones, saponins, and terpenoids.

**Table 4:** Preliminary phytochemical screening of the selected medicinal plants

Phytochemicals	Medicinal plant extracts							
	*A. flagellaris* (root)	*B. antidysenterica* (fruit)	*C. trigyna* (seed, inflorescence)	*C. rueppellii* (root)	*G. involucrata* (root)	*G. involucrata* (stem)	*P*. *steudneri* (pseudobulb)	*S. venosum* (tuber)
Alkaloids	+	+	+	+	–	–	–	+
Anthocyanins	+	–	+	–	+	+	+	–
Anthraquinones	–	–	–	–	+	+	+	–
Cardiac glycosides	+	–	+	+	+	+	+	+
Coumarins	+	+	+	+	–	–	+	+
Flavonoids	+	+	+	–	+	+	+	+
Phenols	+	+	+	–	+	+	+	+
Saponins	+	–	+	–	+	–	–	–
Steroids	–	+	+	–	–	+	+	+
Tannins	+	+	+	–	+	+	+	+
Terpenoids	+	+	+	+	+	+	–	–

(+), present; (–), absent

## Discussion

In the ethnomedicinal perspective, leaves and stems of *A. flagellaris* were reported to be used against gonorrhea and syphilis in Nigeria [23], its fruits for eye diseases, and its roots for measles in Uganda [24]. Leaves of *B. antidysenterica* were used to treat wounds in Zuway Dugda district [25] and diarrhea surrounding the Gullele Botanic Garden in central Ethiopia [26]. Whole parts of *Celosia trigyna* were reported to heal arthritis, diarrhea, and dysentery in Kafa Zone [27], and seeds were used to treat tapeworm in Libo Kemkem district, northwest Ethiopia [28]. Leaves and roots of *Crepis rueppellii* were used to cure dysentery by residents on the Dek Island of Lake Tana, northwest Ethiopia [29]. Roots of *Gnidia involucrata* were reported to treat gonorrhea and ascaris in the Bule Hora district of southern Ethiopia. The tubers of *S. venosum* were traditionally used to treat ascaris [29] and hemorrhoids [30] in northwest Ethiopia. Hence, the literature supports the ethnomedicinal data in the present study and the effectiveness of the study plants against several infectious diseases, except for *P. steudneri*, which has not been studied yet.

In the current study, it was observed that the minimum inhibitory concentrations (MIC) of the investigated medicinal plants were dependent on the types of bacterial strains. The investigated medicinal plants exhibited various MIC values against different gram-negative and gram-positive bacterial strains. Similar to the current findings, the previous studies reported *P. mirabilis* as an antimicrobial-resistant bacterial strain [31, 32]. In line with the present study, other previous studies also reported the antimicrobial resistance of *S. epidermidis* among gram-positive bacteria [33, 34]. This indicates the multiple antibiotic resistance of both *P. mirabilis* and *S. epidermidis*.

An earlier study conducted by Taye et al. [35] reported that the methanolic extracts of *B. antidysenterica* root showed a MIC value of 15.63 mg/mL against *S. aureus*, while the ethanolic extracts of its fruits inhibited the same bacterial strain at a MIC value of 4.00 mg/mL in the present study. Here, variation in the antibacterial activity of *B. antidysenterica* might be due to differences in the extraction solvents used or the bioactivity of the tested plant parts. A similar study conducted by Kalbessa et al. [36] reported the highest efficacy of the ethyl acetate extracts of *G. involucrata* root bark against *S. aureus* compared to the other bacterial strains. However, the current findings revealed the most sensitive bacteria, *S. agalactiae*, to the ethanolic extracts of *G. involucrata* roots than *S. aureus*. On the other hand, the study conducted by Zakerifar et al. [37] showed that *S. agalactiae* was reported to be resistant to certain antibiotics like erythromycin, levofloxacin, ofloxacin, quinupristin, and tetracycline and susceptible to chloramphenicol, gentamicin, linezolid, penicillin, and vancomycin. In this respect, there are variations in the antibacterial efficacy of medicinal plants and differences in the degree of susceptibility of bacterial strains. Thus, MIC values varied among the extracts of different medicinal plants in the present study. This might be linked to the difference in the biologically active phytochemicals they contain [38]. Besides, MIC values differed among different bacterial strains owing to their variation in antibiotic resistance.

The study conducted by Odeja et al. [23] showed that the leaf essential oil of *A. flagellaris* had high antioxidant activity, with 90.74% inhibition of DPPH free radicals at a concentration of 500 µg/mL. In the present findings, however, the ethanolic extracts of its root exhibited 51.28% inhibition at the same concentration. In this case, the variation in the antioxidant activity of *A. flagellaris* might be because of the extraction methods employed or the excess of phytoconstituents in leaves rather than roots. The other study conducted by Kalbessa et al. [36] indicated that ethyl acetate extracts of *G. involucrata* root bark and its isolated compound exhibited 70.70 and 85.80% inhibition at concentrations of 100 µg/mL, respectively. This is slightly comparable with the current findings, in which the ethanolic extracts of *G. involucrata* stem showed 68.91% inhibition at 125 µg/mL. This confirms the antioxidant potential of different parts of *G. involucrata* to reduce risks related to free radicals.

The antibacterial and antioxidant activities of medicinal plants depend on their phytochemical constituents. This is due to the fact that the phytochemical constituents of the medicinal plants are associated with their antioxidant and antibacterial activities [9, 39]. Medicinal plants contain mainly phenolic antioxidants like ß-carotene, flavonoids, phenolic acids, terpenes, tocopherols, vitamin C, and so on [40]. Antioxidant phenolic compounds scavenge free radicals and prevent oxidation of cellular components either by donating hydrogen atoms to free radicals to form stable, harmless compounds [12] or by inhibiting enzymes responsible for the production of reactive oxygen species [11]. Thus, they take part in the prevention or treatment of oxidative stress-related diseases, for example, atherosclerosis, biliary diseases, cancer, dementia, diabetes, hypertension, kidney disease, macular degeneration, neurodegenerative diseases, and obesity [41].

The phytochemical screening of ethanolic extracts showed that the selected medicinal plants contain important phytochemicals, which could play crucial roles in their bioactivities. Phytochemical constituents, mainly bioactive secondary metabolites, play significant roles in the bioactivities of medicinal plants by eliciting a definite and specific action on the human body [42]. The current results showed the presence of steroids in the ethanolic extracts of *B. antidysenterica* fruits, *C. trigyna* inflorescence with seeds, *G. involucrata* stems, *P. steudneri* pseudobulbs, and *S. venosum* tubers. Steroids are used to treat rheumatism, asthma, allergies, skin infections, and inflammations [43] and to relieve inflammation and swelling in cancer patients [42, 43]. Cardioactive steroids, for example, cardenolides, improve heart function, although they are highly toxic and received at a therapeutic dose of 60% of the lethal dose [44]. Results of phytochemical screening revealed the presence of alkaloids in the extracts of *A. flagellaris*, *B. antidysenterica*,* C. trigyna*,* C. rueppellii*, and *S. venosum*. Isoquinoline alkaloids are found in higher plants and are known to have antispasmodic, antiviral, antifungal, anticancer, antioxidant, and enzyme inhibitory activities [45]. Besides, diterpenoid alkaloids are potent to treat various cancers as new drugs [46].

Flavonoids, tannins, and phenols were identified from the ethanolic extracts of all investigated medicinal plants except that of *C. rueppellii*. Flavonoids have antioxidant, anti-inflammatory, and antimicrobial activities; hence, they attribute to the medicinal properties of different medicinal plants [43]. For instance, plants like *Zingiber*, *Curcuma*, and *Acorus* were reported as sources of antibacterial and antiseptic agents owing to their flavonoid content [47]. Tannins were described as healing agents for inflammation, hemorrhoids, and gonorrhea [42] and were known to have anticancer [47] and antidiabetic [48] effects. Polyphenolic compounds have been beneficial as antioxidants, anti-inflammatory, and antibacterial agents and reduce blood pressure and heart disease [47]. Out of the examined plants, saponins were found in extracts of *A. flagellaris* roots, *C. trigyna* inflorescence with seeds, and *G. involucrata* roots. Saponins were stated to treat different human diseases, such as skin infections, liver diseases, trauma, chronic venous insufficiency, and kidney diseases [49].

The tested medicinal plants were positive for cardiac glycosides, except for *B. antidysenterica*. In agreement with the current findings, the studies conducted by Liu et al. [50] and Ravi et al. [51] reported the existence of cardiac glycosides in many medicinal plants. Cardiac glycosides have beneficial effects for the heart [47] in that they treat congestive heart failure and cardiac arrhythmia by inhibiting the Na^+^/K^+^ pump and increasing the level of calcium ions (Ca^+^), which enhances the contraction of heart muscles and reduces swelling [13]. Terpenoids were detected in ethanolic extracts of *A. flagellaris* roots, *B. antidysenterica* fruits, *C. trigyna* inflorescence with seeds, *C. rueppellii* roots, and *G. involucrata* roots and stems. Medicinally, they provide significant actions such as antiviral, antibacterial, antimalarial, anti-inflammatory, anticancer, and inhibition of cholesterol synthesis [42]. Coumarins are among the essential phytochemical compounds found in medicinal plants [42]. In this regard, results from the current study indicated the presence of coumarins in ethanolic extracts of the investigated medicinal plants, except in the roots and stems of *G. involucrata*. Medicinally, coumarins were appreciated to treat microbial infections, cancers, tuberculosis, inflammatory diseases, malaria, and AIDS-acquired immunodeficiency syndrome [52].

The qualitative phytochemical screening revealed that anthraquinones were identified from ethanolic extracts of *G. involucrata* roots and stems and *P. steudneri* pseudobulbs. The plant-derived natural anthraquinones were reported to have antiviral potential against different infectious viruses [53]. Results also showed that the examined medicinal plants, such as *A. flagellaris, C. trigyna, G. involucrata, and P. steudneri*, were positive for the anthocyanins test. Plant-based anthocyanins have antioxidant properties that play important roles in health and therapeutic effects [54]. Furthermore, the presence of phytochemicals such as alkaloids, flavonoids, glycosides, phenolic compounds, saponins, tannins, and triterpenoids promotes the anthelmintic properties of medicinal plants [55].

In the present study, a comparative investigation was carried out between the extracts of *G. involucrata* roots and stems, though local people traditionally use its roots. The extract of *G. involucrata* stems showed even higher antibacterial activity, antioxidant capacity, and phytochemical contents than the root extract. This might be due to the distribution of secondary metabolites from the areas of synthesis (leaves) to the areas of sink (roots and stems) *via* phloem tissue. Hence, results from the current study suggest the use of *G. involucrata* stems instead of its roots since root harvesting is usually destructive for the sustainable use of medicinal plants.

## Conclusions

Ethanolic extracts of the investigated medicinal plants were active against different gram-negative and gram-positive bacterial strains at various concentrations. Additionally, the ethanolic extracts exhibited considerable antioxidant activity compared to ascorbic acid. The qualitative phytochemical screening revealed the presence of important bioactive compounds in the tested medicinal plants. Hence, the findings from this study support the traditional medicinal use of the investigated plants. The study was restricted to their 80% ethanolic extracts. Thus, further investigations using different solvents of various polarities will be required to extract lead compounds for the development of appropriate drugs. Besides, toxicity studies will be necessary to encourage their further use.

## Data Availability

The data that support the findings reported herein are available from the corresponding author upon reasonable request.

## Abbreviations

ATCC: American type culture collection
DPPH: 2,2-diphenyl-1-picrylhydrazyl
FL: Fidelity level
IC_50_: 50% inhibition concentration
MIC: Minimum inhibitory concentration
PCA: Principal Component Analysis
UV-Vis: Ultra-Violet Visible
